# Magnetic multi-granule nanoclusters: A model system that exhibits universal size effect of magnetic coercivity

**DOI:** 10.1038/srep12135

**Published:** 2015-07-17

**Authors:** Ji Sung Lee, Jin Myung Cha, Ha Young Yoon, Jin-Kyu Lee, Young Keun Kim

**Affiliations:** 1 Department of Materials Science and Engineering, Korea University, 145 Anam-ro, Seongbuk-gu, Seoul 136-713, Korea; 2 Department of Chemistry, Seoul National University, 1 Gwanak-ro, Gwanak-gu, Seoul 151-742, Korea

## Abstract

It is well known that the coercivity of magnetic nanomaterials increases up to a maximum and then decreases to zero with decreasing particle size. However, until now, no single synthesis method has been able to produce magnetic nanoparticles with a wide range of sizes, i.e., from 10 to 500 nm, in order to uncover the coercivity evolution. Here we report the characterization of magnetite (Fe_3_O_4_) multi-granule nanoclusters (MGNCs) to demonstrate the transitional behaviour of coercivity. The *M–H* curves indicate that our samples had a relatively high saturation magnetization (*M*_S_) value of ~70 emu/g and that the coercivity (*H*_c_) increased to the maximum value of ~48 Oe until the nanoclusters reached a size of ~120 nm; the coercivity then gradually decreased to zero.

Magnetic nanoparticles have been of great interest for researchers in various fields, including biological biomedicine^1^,^2^. The finite-size effect of magnetic nanoparticles, which differentiates them from bulk materials, influences their physicochemical and pharmacokinetic properties and is therefore a critical parameter^3^,^4^. Superparamagnetic nanoparticles are preferred in biomedical applications because they have zero net magnetization at room temperature (RT) and thus do not agglomerate. In the past, magnetic properties of superparamagnetic magnetite colloidal nanocrystal clusters prepared via a hydrolysis reaction were reported where the cluster size was varied from 30 to 180 nm, consisting of nanocrystals with a size of ~8 nm^5^. Nevertheless, the superparamagnetic particle systems, in general, require a relatively large external magnetic field in order to obtain a magnetic response. In light of this, ferromagnetic nanoparticles capable of being dispersed in water or phosphate buffer solution (PBS) upon the removal of the magnetic field would be attractive for use in biomolecular separation and purification processes. However, very small nanoparticles can also result in a low saturation magnetization or nonspecific uptake, which reduces their usefulness. Thus adequate control of their size and understanding the changes in magnetic properties as a function of nanoparticle size are crucial.

Unlike saturation magnetization, which is, in principle, size-independent, the coercivity is very sensitive to the size variation. For example, as presented in Fig. 1, it is a well-known fact that the coercivity of a particle gradually increases to a maximum value at a particular size and then rapidly decreases to zero as the particle size further decreases^6^. It is understood that when the particle diameter (*D*) decreases, a magnetic multi-domain (MD) state converts to a single-domain (SD) state. The diameter at which this conversion from MD to SD state occurs is represented as *D*_S_. For an SD particle, the coercivity value is usually high, meaning that it is more difficult to reverse its magnetization. Thus one needs to apply a magnetic field larger than the demagnetization field of the SD particle. The decrease of coercivity below *D*_S_ is due to an increased thermal contribution, which randomizes the magnetization. At an extreme case where the particle size decreases further, the coercivity becomes zero, reaching a superparamagnetic state. The zero-coercivity diameter is denoted as *D*_P_ . Unlike the transition from ferromagnetic to ordinary paramagnetic properties in bulk magnets, the ferromagnetic-to-superparamagnetic transition in fine particles is solely due to the size effect.

However, the coercivity transition behaviour for a wide range of particle sizes, say, from 10 to 500 nm, has rarely been reported. The difficulty appears to be that there has been no single chemical synthesis method capable of producing magnetic nanoparticles over a wide range of diameters uniformly. We recently synthesized magnetite (Fe_3_O_4_) multi-granule nanoclusters (MGNCs) of various sizes by the hydrothermal polyol process and investigated the formation mechanism of MGNCs from the solid-state phase transformation of iron oxides^7^,^8^. A nanocluster consists of many nanogranules, where the spins in the nanogranules are magnetically-coupled. Here we report the transitional behaviour of coercivity for a wide range of nanoparticle sizes by employing MGNCs as a model system.

## Results

**Figure 2:**
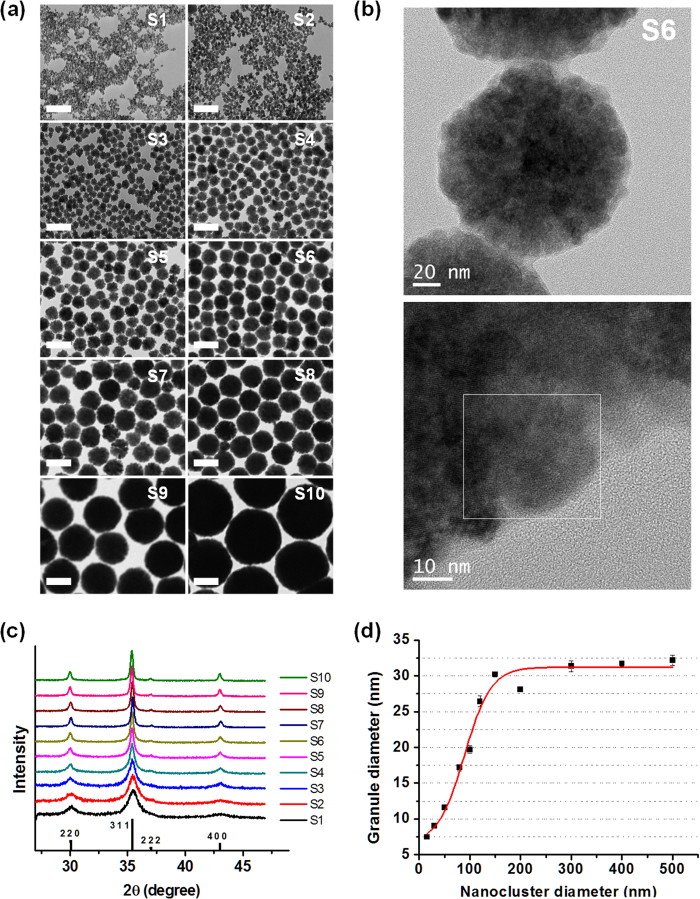
A collection of nanocluster and granule sizes for samples used in this study. (**a**) TEM images of magnetite MGNCs with various diameters (scale bars in all images are 200 nm), (**b**) high-resolution TEM images of sample S6 (top image shows an isolated MGNC, whereas the bottom image shows a corresponding granule marked in a white box), (**c**) XRD patterns, and (**d**) average granule diameters calculated from the full-width-at-half-maximum (FWHM) of the strongest (311) XRD peaks.

The MGNCs were synthesized *via* a hydrolysis condensation and reductive polyol process^7^. As revealed by transmission electron microscopy (TEM) imaging, the MGNCs were nearly spherical in shape, with diameters ranging from 15 to 500 nm (Fig. 2). The X-ray diffraction (XRD) patterns show the single-phase cubic inverse-spinel structure of Fe_3_O_4_ (magnetite, JCPDS File No. 19-0629). The granule sizes in all MGNCs were calculated by Debye–Scherrer’s equation using the XRD patterns in Fig. 2. The calculation results are given in Table 1.

With respect to the magnetism of our model system, two main questions can be raised: *i) Are these magnetic nanoclusters ferromagnetic or superparamagnetic? and ii) Are there any variations in coercivity associated with the size change?* For particles of a fixed size, the magnetization becomes unstable above the blocking temperature (*T*_B_), which can be estimated as


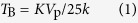


where *K* is the magnetic anisotropy constant (for magnetite, 1.40 × 10^4^ J/m^3^), *V*_P_ the volume, and *k* the Boltzmann constant. For example, from equation (1), the particles with *D*_P_ values of 10 and 20 nm will correspond to *T*_B_ values of 21 and 170 K, respectively. However, when there is a particle-size distribution, the blocking behaviour becomes more complicated because relatively smaller particles tend to be blocked, whereas the other particles remain unblocked, even at the same temperature.

The second question is related to an understanding of the size effect in our samples. The particle size-dependent *H*_c_ behaviour can be expressed as in equation (2)^6^.


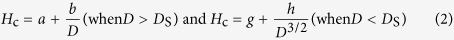


where *a*, *b*, *g*, and *h* are constants. According to Sharrock’s time-dependent *H*_*c*_ expression^9^, *H*_*c*_ decreases inversely proportional to the square root of particle volume (*V*^−1/2^) from the anisotropy field (*H*_o_) value. This *V*^−1/2^ decrease is relevant to the *D*^−3/2^ decrease in Eq. (2) because the energy barrier for switching decreases as the magnetic volume (or particle size) decreases. *D*_S_ can be estimated for a spherical particle with a small anisotropy constant, which fits to our case as represented in equation (3)^10^.


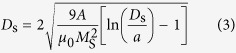


where *μ*_o_ is the magnetic permittivity constant in vacuum (1.26 × 10^−6^ H/m), *M*_S_ the saturation magnetization of MGNCs (3.63 × 10^5^ A/m or 70 emu/g in our measurement), *A* the exchange stiffness (1.33 × 10^−11^ J/m), and *a* the lattice constant of magnetite (8.39 × 10^−10^ m). The solution of equation (3) yields the estimated *D*_S_ for an MD to SD transition of ~105.4 nm.

**Figure 3:**
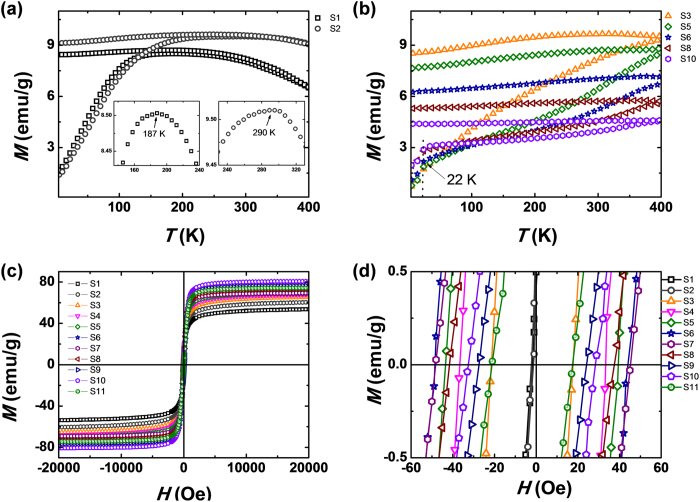
Magnetic property measurements for MGNC samples of various diameters. (**a**) and (**b**) Temperature-dependent magnetization (M–T) curves obtained by ZFC/FC processes measured by PPMS. The top portion of each curve corresponds to the FC process, whereas the bottom of each curve corresponds to the ZFC process. In these subfigures, (**a**) shows superparamagnetic responses, with a *T*_B_ of 187 K and 290 K for S1 and S2, respectively, whereas (**b**) represents ferromagnetic behaviour. (**c**) Magnetic hysteresis curves of MGNC samples measured by VSM at room temperature. Except for the superparamagnetic samples (S1 and S2), all other ferromagnetic samples show *M*_S_ above 70 emu/g. (**d**) Low-field portion showing *H*_C_ values.

Figure 3(a,b) display a collection of zero-field-cooling (ZFC)/field-cooling (FC) *M–T* curves obtained under a magnetic field of 100 Oe for the MGNCs. Fig. 3(a) shows that S1 and S2 are superparamagnetic, whereas the other samples are ferromagnetic at RT. For the superparamagnetic samples, *T*_B_ is visible as the cusp value in the ZFC magnetization curve, which is 187 K for S1 and 290 K for S2. From equation (1), the measured *T*_B_ of 187 K corresponds to *D*_P_ of 20.6 nm and *T*_B_ of 290 K corresponds to *D*_P_ of 23.9 nm. This discrepancy is due to the presence of a particle distribution, as mentioned before. The ZFC curves of Fig. 3(b) show other specific temperatures (near 20 K) where the magnetization abruptly decreases. This phenomena is probably related to the Verwey transition^11^ that occurs at 120 K (=*T*_V_) for bulk magnetite as a result of charge ordering, in which the valence of the Fe ions changes^15^. Our samples show distinctively low *T*_V_ values as compared with their bulk counterparts, despite having sufficiently large clusters. Therefore, the most likely factor affecting *T*_V_ is not necessarily the size of the cluster but the size of the granules within the cluster.

A collection of hysteresis curves (*M–H* responses) measured by vibrating sample magnetometry (VSM) at RT is depicted in Fig. 3(c,d). The figures indicate that our samples have relatively high *M*_S_ values of ~70 emu/g (the *M*_S_ of bulk magnetite is 91 emu/g) for ferromagnetic MGNCs with *D* ≥ 80 nm. The *M*_S_ value of the MGNCs is approximately 22% smaller than the bulk one. When *D* < 50 nm, the *M*_S_ value is reduced due to an increase in the surface area/volume ratio, causing spin canting at the surface^12^.

**Figure 4:**
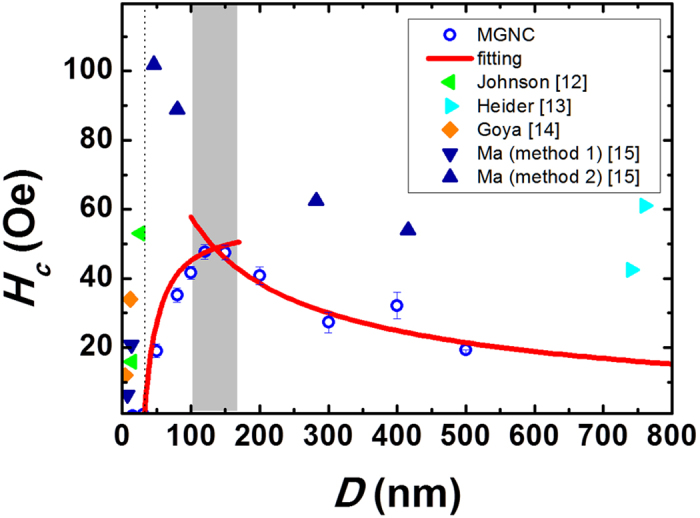
Variation of coercivity *H*_C_ as a function of particle diameter *D.* Note that the magnetite nanoparticles used in other studies appear to have size distribution, and the shape of the particles with sizes over 40 nm is relatively nonspherical. These contributions resulted in higher *H*_c_ values.

Figure 4 displays the *H*_c_ variation of MGNCs as a function of diameter *D*^13^,^14^,^15^,^16^. To the best of our knowledge, this is the first report of *H*_c_ variation as a function of a wide range of particle sizes (0 < *D* < 500 nm) for magnetite nanoparticles synthesized by the same method. A selection of *H*_c_ values collected from other studies is included for reference purposes. Our samples possess magnetic softness, as evidenced by relatively low *H*_c_ values, implying that our samples have fewer defects and a uniform shape. As mentioned previously, the value of *H*_c_ increased to the maximum value (in our case ~48 Oe) for S6 and then decreased until it reached the superparamagnetic limit. The curve-fitting according to equation (2) yields fitting parameters of *a* = 8, *b* = 6,000, *g* = 55, and *h* = −10,000. For each sample, we used an average value after three hysteresis measurements. Based upon the analysis of equation (3), we estimated the value of *D*_S_ to be 105.4 nm, which is very close to the measured value, 123 nm, of S6. This result indicates that the crystallites inside an MGNC are magnetically-coupled with each other, and thus one MGNC behaves like a single spherical particle.

In summary, magnetic properties of Fe_3_O_4_ MGNCs with diameters ranging from 10 to 500 nm were investigated by both theoretical models and experimental measurements. The temperature-dependent magnetization measurement results showed that samples with diameters of 16 and 32 nm were superparamagnetic, whereas the other larger samples were ferromagnetic. Based upon the magnetic hysteresis measurements at RT, we were able to monitor the entire spectrum of coercivity variations for a wide range of particle sizes.

## Methods

### Chemicals

FeCl_3_·6H_2_O (>97%, Sigma-Aldrich, Korea), sodium acetate (>98.5%, Samchun Chemicals, Korea), and ethylene glycol (>99.5%, Samchun Chemicals, Korea) were obtained from commercial sources and used as received.

### Synthesis of magnetite multi-granule nanoclusters (MGNCs)

In a typical synthesis of MGNCs, FeCl_3_·6H_2_O, sodium acetate, and distilled water were completely dissolved in ethylene glycol by vigorous mechanical stirring, and then the solution was refluxed for several hours. After cooling down to room temperature, the MGNCs were separated magnetically and washed with ethanol and distilled water several times to eliminate organic and inorganic byproducts.

### Structural characterization

The morphology of the MGNCs was analysed using transmission electron microscopy (TEM, Hitachi-7600) operated at an accelerating voltage of 100 kV. The detailed structure and crystallinity of the individual MGNCs were determined by analytical TEM (PEI Tecnai F20) at 200 kV. Samples for TEM experiments were prepared by diluting washed samples in absolute ethanol or distilled water, and then a drop of the sample solution was placed on a carbon-coated copper grid and silicon wafer. The sizes of the nanoclusters were counted in the TEM images, and they were found to follow a Gaussian distribution (see Fig. S1). The crystal structure and crystallite size of the MGNCs were determined using powder X-ray diffraction (XRD, MAC Science M18XHF-XRA) with Cu Kα radiation (λ = 1.5406 Å). An accelerating voltage of 40 kV and an emission current of 200 mA were used. Scans were recorded for 2*θ* values between 20° and 80° with a scanning speed of 5° min^−1^ (1° min^−1^ for determination of the average crystallite size). The average crystallite size was calculated by the Debye–Scherrer equation, *D*_xrd_* = *0.9 λ*/β*cos*θ*, where *D*_xrd_ is the average crystal diameter, λ the wavelength of the X-ray employed, *β* the full-width-at-half-maximum (FWHM) of the strongest peak (3 1 1) in radians, and *θ* the Bragg angle.

### Magnetic property characterization

Magnetic properties were analysed in powder samples by measuring zero-field-cooling (ZFC)/field-cooling (FC) curves and magnetization curves [*M–H*] using a physical property measurement system (Quantum Design, PPMS 6000) equipped with a vibrating sample magnetometer (P525) and a vibrating sample magnetometer (Micro Sense, EV9). For the ZFC/FC measurements, a sample was cooled down to 4 K in a zero applied field from RT, and the magnetization was measured stepwise with increasing temperature until reaching 400 K in a field of 100 Oe (ZFC). Then the sample was cooled to 4 K under the same field and the magnetization was measured by the same process. *M–H* was measured at 300 K with an applied field of up to 30 kOe (see Fig. S2).

## Additional Information

**How to cite this article**: Lee, J. S. *et al.* Magnetic multi-granule nanoclusters: A model system that exhibits universal size effect of magnetic coercivity. *Sci. Rep.* **5**, 12135; doi: 10.1038/srep12135 (2015).

## Supplementary Material

Supplementary Information

## Figures and Tables

**Figure 1:**
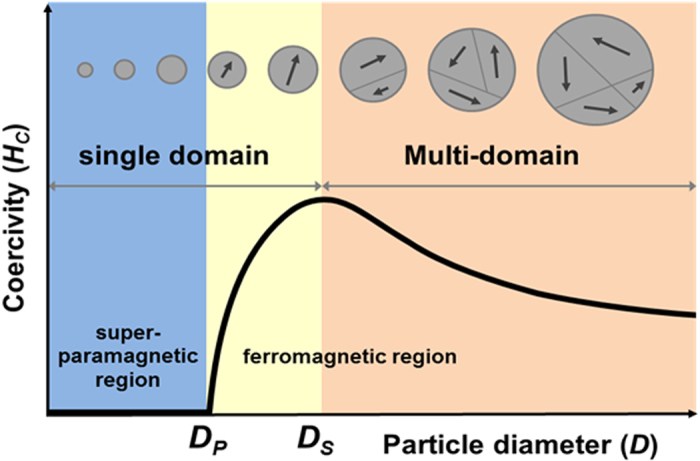
A schematic showing the coercivity (*H*_c_) behaviour of a magnetic particle as a function of its diameter (*D*). As a particle size decreases, the domain wall disappears, resulting in an increase in *H*_c_ until the particle size reaches *D*_S_. When the particle size is further decreased, thermal agitation energy overcomes magnetic anisotropy energy and, as a result, the particle enters the superparamagnetic regime, *D*_P_.

**Table 1:** Nanocluster and granule diameters analysed from TEM images and XRD patterns.

Sample	Nanocluster diameter [nm]	Granule diameter [nm]	Coercivity [Oe]	Magnetization at 20 kOe [emu g^−1^]
S1	16	7.5	0.25	53.8
S2	32	9.0	0.57	60.4
S3	53	11.6	19.1	65.3
S4	80	17.2	35.1	67.8
S5	99	19.7	41.6	69.5
S6	123	26.4	47.7	70.5
S7	152	30.2	47.5	68.7
S8	181	28.1	40.8	72.7
S9	278	31.4	27.3	76.3
S10	422	31.8	32.2	80.4
S11	512	32.2	19.4	74.0

The granule diameters were calculated by Debye-Scherrer’s equation. Coercivity and magnetization values were measured by VSM at RT.
