# Supplementary material for: Magnetic multi-granule nanoclusters: A model system that exhibits universal size effect of magnetic coercivity
Source: Sci Rep. 2015 Jul 17;5:12135. doi: 10.1038/srep12135 (PMC4505357; doi:10.1038/srep12135)
Supplement: Supplementary Information [file srep12135-s1.pdf]

Supplementary Information

Magnetic multi-granule nanoclusters: A model system that exhibits universal size effect of magnetic coercivity

Ji Sung Lee[1,]*, Jin Myung Cha[2,]*, Ha Young Yoon[1], Jin-Kyu Lee[2,] and Young Keun Kim[1,]

[1] Department of Materials Science and Engineering, Korea University, 145 Anam-ro, Seongbuk-gu, Seoul 136-713, Korea

[2] Department of Chemistry, Seoul National University, 1 Gwanak-ro, Gwanak-gu, Seoul 151-742, Korea

*These authors contributed equally this work.

Correspondence and requests for materials should be addressed to Y.K.K. (email: ykim97@korea.ac.kr) or J.-K.L. (jinklee@snu.ac.kr)

Supplementary Data

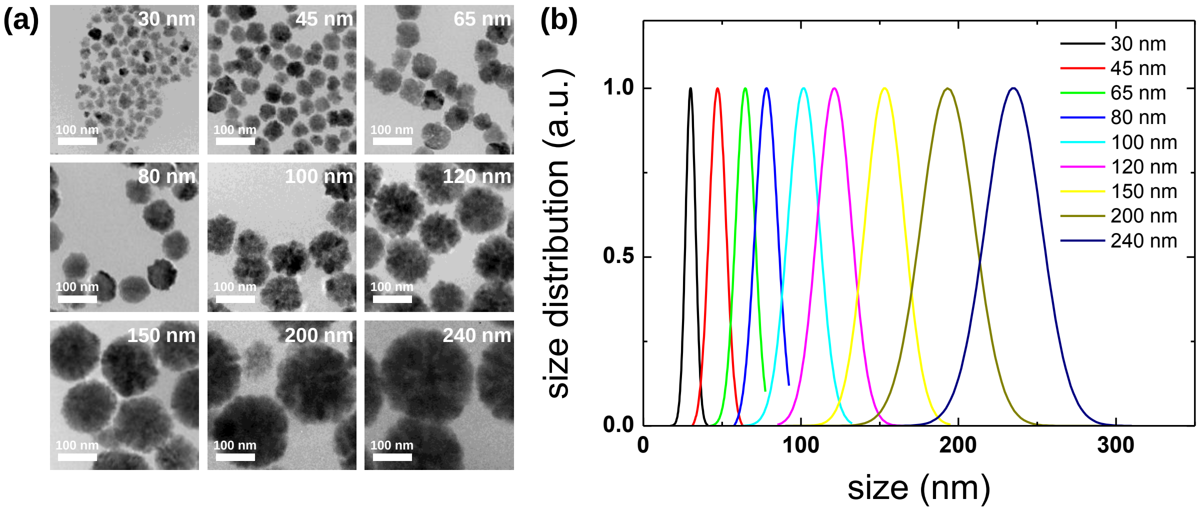

Figure S1. Size distribution of MGNCs. The sizes of the nanoclusters were counted in the TEM images and they follow a Gaussian distribution. Size counting reveals that the nanoclusters have averaged diameters and standard deviation (σ) values of 30 ± 3 nm, 42 ± 4 nm, 64 ± 6 nm, 78 ± 7 nm, 108 ± 7 nm, 125 ± 8 nm, 153 ± 12 nm, 193.2 ± 17 nm, and 231 ± 15 nm.

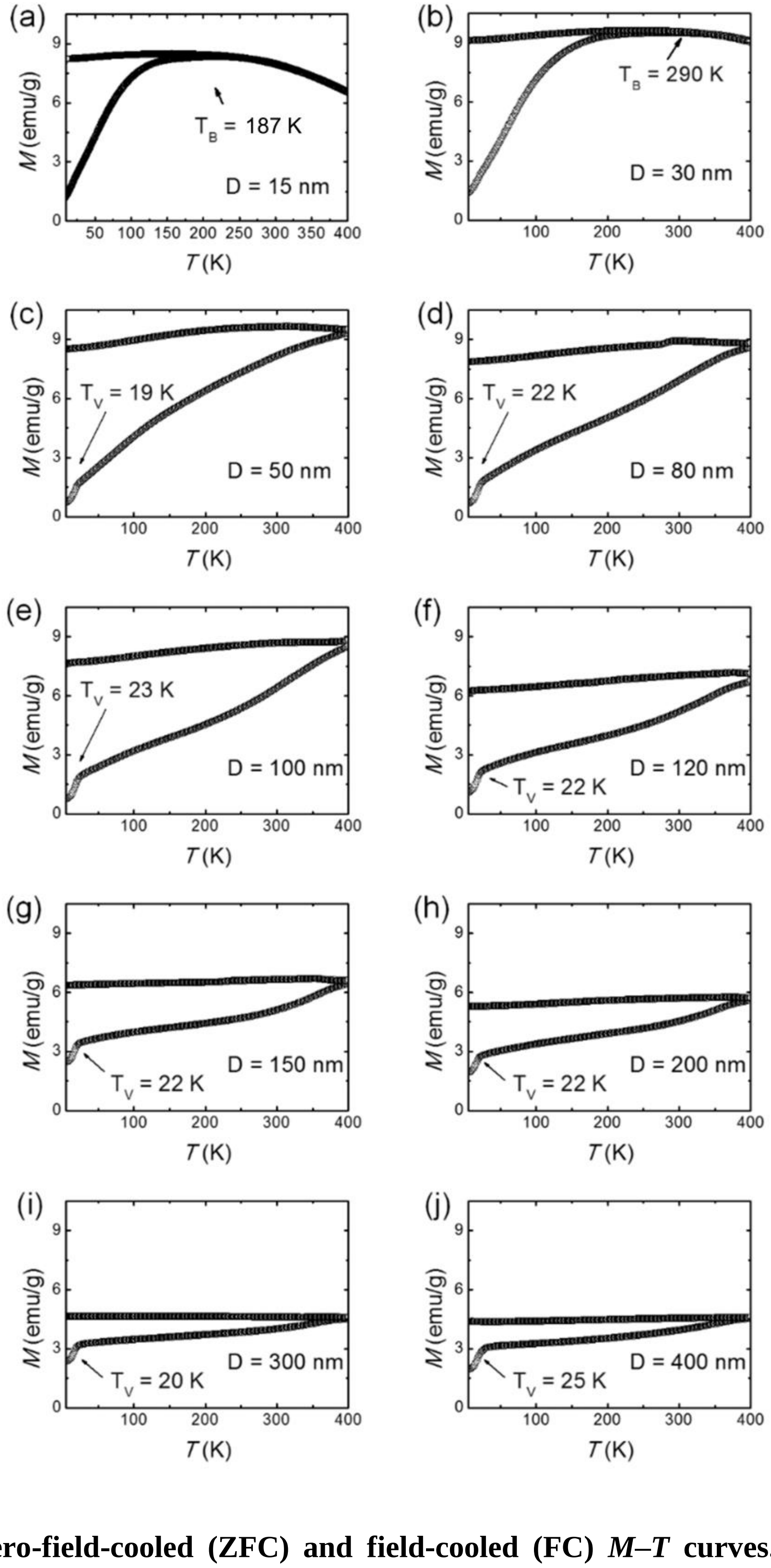

Figure S2. Zero-field-cooled (ZFC) and field-cooled (FC) *M*–*T* curves. All data were obtained from the PPMS measurements under magnetic field of 100 Oe.
